# Circulating Tumor Cells in Breast Cancer Patients: A Balancing Act between Stemness, EMT Features and DNA Damage Responses

**DOI:** 10.3390/cancers14040997

**Published:** 2022-02-16

**Authors:** Benedikt Heitmeir, Miriam Deniz, Wolfgang Janni, Brigitte Rack, Fabienne Schochter, Lisa Wiesmüller

**Affiliations:** Department of Obstetrics and Gynecology, Ulm University, 89075 Ulm, Germany; benedikt.heitmeir@uniklinik-ulm.de (B.H.); miriam.deniz@uniklinik-ulm.de (M.D.); direktion.frauenklinik@uniklinik-ulm.de (W.J.); brigitte.rack@uniklinik-ulm.de (B.R.); fabienne.schochter@uniklinik-ulm.de (F.S.)

**Keywords:** cancer stem cell, circulating tumor cells, DNA damage response, epithelial-mesenchymal transition, metastasis

## Abstract

**Simple Summary:**

Circulating tumor cells dissociate from the primary tumor, enter the bloodstream and travel to distant sites where they seed metastases. To endow these tumor cells with the features necessary for this journey, they must undergo dramatic shape changes, acquire migratory potential, alter their metabolism, and quickly adapt to insults in each new environment. To permit such phenotypic changes in multiple directions, they often acquire a more primitive state reminiscent of stem cells in the embryo. These changes are coupled with altered capacities and qualities to remove DNA lesions such as those induced by a metabolic shift or an immune cell attack. Defects in DNA repair cause mutations, leading to hereditary breast cancer and accelerating progression. Enhanced DNA repair causes resistance to chemotherapeutic treatment. Therefore, it is of utmost interest to understand the choreography of these functions in circulating tumor cells at the molecular level, because they represent targets to fight chemoresistant metastases.

**Abstract:**

Circulating tumor cells (CTCs) traverse vessels to travel from the primary tumor to distant organs where they adhere, transmigrate, and seed metastases. To cope with these challenges, CTCs have reached maximal flexibility to change their differentiation status, morphology, migratory capacity, and their responses to genotoxic stress caused by metabolic changes, hormones, the inflammatory environment, or cytostatic treatment. A significant percentage of breast cancer cells are defective in homologous recombination repair and other mechanisms that protect the integrity of the replication fork. To prevent cell death caused by broken forks, alternative, mutagenic repair, and bypass pathways are engaged but these increase genomic instability. CTCs, arising from such breast tumors, are endowed with an even larger toolbox of escape mechanisms that can be switched on and off at different stages during their journey according to the stress stimulus. Accumulating evidence suggests that DNA damage responses, DNA repair, and replication are integral parts of a regulatory network orchestrating the plasticity of stemness features and transitions between epithelial and mesenchymal states in CTCs. This review summarizes the published information on these regulatory circuits of relevance for the design of biomarkers reflecting CTC functions in real-time to monitor therapeutic responses and detect evolving chemoresistance mechanisms.

## 1. DNA Repair Defects Play Key Roles during the Development of Breast Cancer

Primary tumor cells are genomically unstable, which exacerbates during tumor evolution towards CTCs through a fatal combination of aberrant cell cycle checkpoints and DNA damage responses causing replication stress [1,2]. Persistent replication stress increases the likelihood of the cleavage of stalled replication forks, and the resulting one-ended DNA double-strand breaks (DSBs) can be repaired by homologous recombination (HR) [3]. HR is mostly error-free, but defective in roughly one third of breast cancers (BCs) [4]. To evade DSB-induced cell death, these cells have to use alternative error-prone DSB repair pathways such as single-strand annealing (SSA), microhomology-mediated end joining (MMEJ) and related activities at replication forks, such as break-induced replication [5,6,7]. This pathway shift leads to simultaneous formation of copy number alterations (CNAs) in the genome [2,8] driving neoplastic development and tumor progression [9,10,11]. Many factors inactivated in BC, including BRCA1, BRCA2, ATM, BARD1, BRIP1, CHK1, CHK2, ABRAXAS1, MRE11, Nibrin, PALB2, RAD50, RAD51C and RAD51D, are involved in the repair of DSBs by HR [12]. Beyond their DNA repair functions, BRCA1 and BRCA2 protect stalled replication forks from nucleolytic attack, i.e., from the accumulation of DNA damage already before repair [13,14]. Accordingly, BRCA1/2-pathway deficient cells show a high sensitivity to cytostatics generating DNA replication blocks, such as crosslinking platinum derivatives, and drugs interfering with unperturbed fork progression; for example, poly (ADP-ribose) polymerase (PARP)1 inhibitors [12,15].

Why is an intact HR pathway required to prevent tumorigenesis in the mammary gland? A key finding to answer this question was made by Stork and colleagues [16]. These authors demonstrated an increased formation of R-loops, i.e., transcriptional RNA-DNA intermediates, by the hormone estrogen, because it mediates transcription of estrogen-responsive genes in breast tissue. These R-loops seem to be the main cause of DSBs in response to the hormone and colocalize with genomic rearrangement sites. BRCA1 itself is recruited to R-loops, where it mitigates R-loop and DNA damage accumulation and ultimately insertion and deletion mutations associated with *BRCA1*-mutated BC [17,18]. Intriguingly, this R-loop preventive effect of BRCA1 contributes to breast epithelial cell differentiation from luminal progenitor to mature luminal cells, which can explain why *BRCA1*-mutated basal-like BC originates from more primitive luminal progenitor cells [17]. Basal-like BC is closely related to so-called triple-negative BC (TNBC), frequently featuring epithelial-mesenchymal transition (EMT) [19]. EMT is crucial for the release of CTCs with metastasizing potential and modifies therapeutic responses [20]. This review explores the current state of the research dissecting the complex and dynamic molecular network orchestrating EMT, stemness features and DNA repair in CTCs from metastatic BC (MBC) patients.

## 2. Dynamic Changes in DNA Damage Responses Are Intricately Linked with EMT and Stemness Features during Breast Cancer Progression

Inactivation of the BRCA1/BRCA2-dependent HR pathway has been connected with hereditary BC and the sporadic form of TNBC, the latter of which makes up approximately 10–20% of BC cases. Thus, deleterious mutations and/or reduced expression of HR genes were frequently observed in TNBC [21,22,23]. For TNBC, defined by estrogen and progesterone receptor negativity (ER-, PR-) and a lack of HER2 overexpression (HER2-), prognosis is poor and targeted therapies are limited [24,25,26]. Chemotherapy of TNBC and MBC patients still mostly relies on cytostatics such as taxanes and anthracyclines, but combinations with novel compounds targeting immune checkpoints [27,28] or HR-defects have shown promise. The BrighTNess phase 3 trial for TNBC patients compared different drug combinations including Carboplatin and a PARP inhibitor [15]. Thus, 158 patients were treated with Paclitaxel, 160 cases with Paclitaxel plus Carboplatin, and 316 cases with Paclitaxel plus Carboplatin plus the PARP inhibitor Veliparib. This trial identified platinum derivatives as an additional treatment option without the further advantage of using the PARP inhibitor. On the other hand, two phase 3 trials, OLYMPIAD and EMBRACA, revealed improved PFS of MBC patients with *BRCA1*/*2*-mutations following treatment with PARP inhibitors versus chemotherapy [29,30]. Accordingly, in Germany, platinum derivatives are recommended for chemotherapy of TNBC patients regardless of the *BRCA1/2* status, and the well-tolerated PARP inhibitors Olaparib and Talazoparib for MBC patients with germline *BRCA1*/*2* mutations [29,30,31].

**Figure 1:**
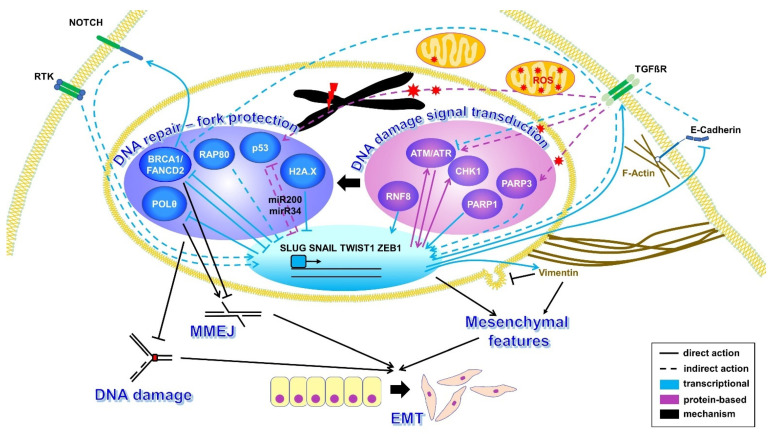
Crosstalk between DNA damage responses and epithelial-mesenchymal transition (EMT) signaling pathways in breast cancer (BC) cells. The transcription factors SLUG, SNAIL, TWIST1 and ZEB1 regulate the expression of multiple factors inducing EMT. In particular, mesenchymal features are induced by the loss of the cell–cell adhesion molecule E-Cadherin and elevated expression of the intermediate filament Vimentin, blocking nuclear rupture. EMT transcription factor-inducing signals are transmitted from the cellular surface by tyrosine kinase receptors (RTKs), NOTCH and TGFßR. TGFß-signaling also activates ATM, p53 and PARP3 via reactive oxygen species (ROS) but also inhibits expression of BRCA1 and ATM. The DNA damage sensing proteins, ATM, ATR, CHK1, PARP1, PARP3 and RNF8, transducing the signal through post-translational protein modifications, promote EMT transcription factors with positive feedback on ATM. Conversely, several proteins involved in DNA repair and/or the protection of DNA replication forks (BRCA1, FANCD2, RAP80, p53, H2A.X) inhibit EMT-inducing transcription factors with negative feedback on the repair proteins polymerase θ, BRCA1 and p53. Accumulation of DNA damage such as that caused by a failure to protect forks and/or to repair them in BRCA1-mutated BC cells triggers EMT. HR dysfunction in these cells de-represses polymerase θ and, therefore, mutagenic repair by microhomology-mediated end joining (MMEJ), which rescues the survival of these cells. For further details, references and abbreviations see Section 2.1 in the main text and Table 1.

More than a third of TNBC patients develop distant metastases through hematogenous spread of the MBC cells [26,32]. TNBC cells frequently display EMT, i.e., an acquisition of mesenchymal and a loss of epithelial cell characteristics (see Figure 1), which plays a crucial role in the release of CTCs with metastasizing potential and modulates the therapeutic response [33]. The high mutational burden of MBC [26] suggests DNA repair and replication abnormalities in CTCs. Therefore, a better understanding of the biology of CTCs affecting these DNA damage responses may provide clues to the development of individually tailored therapies for MBC patients.

### 2.1. EMT and DNA Repair

EMT plays an important part in early embryogenesis and wound healing. It is the main process underlying neural crest formation, as well as several other differentiation processes, as, e.g., mesoderm formation. Since the scope of this biological principle is too broad to be covered completely, we would like to refer the reader to comprehensive reviews such as that presented by Yang and Weinberg [20]. Although this process plays a physiological role during development in the early fetal period, it is hijacked by epithelial tumor cells to acquire mesenchymal features [20]. During this (epithelial-mesenchymal) transition, the cancer cells lose their polarity and organization, typical of epithelial phenotypes, but gain the ability to enter the bloodstream and disseminate.

#### 2.1.1. Signaling from the Cellular Surface

The degree of E-Cadherin expression determines the manifestation of the luminal epithelial phenotype in BC cells [62]. Mesenchymal differentiation can be triggered by extracellular factors. This will ultimately downregulate E-Cadherin to loosen cell–cell contacts and upregulate Vimentin to induce the morphological changes of migrating tumor cells [63]. Important extracellular stimuli are given by the cytokine TGFß, controlling transcription via SMAD proteins [64] and JAGGED-NOTCH signaling [36] as well as ligands of receptor tyrosine kinases (RTK), which all lead to an induction of SLUG (SNAI2), SNAIL (SNAI1), TWIST1 and/or ZEB1 [34]. These transcription factors and master EMT regulators downregulate the determinants of epithelial cells, E-Cadherin in particular, and upregulate the executers of EMT, i.e., cytoskeletal proteins, such as the intermediate filament Vimentin [43,44,45,65]. These changes lead to a loss of adhesion and other epithelial cell characteristics, mesenchymal differentiation, and ultimately CTC spread and metastasis [20,43,66,67]. According to the CTC-theory, also known as the “Seed and Soil” theory, the tumor cells are then no longer bound to their site of origin, move into the bloodstream, and spread to distant locations where they revert to their original epithelial phenotype (MET) to permit metastatic colonization [68,69].

Several genetic changes facilitate EMT. Both hereditary and acquired mutations were described in *CDH1*, causing infiltrative lobular BC [70,71,72]. *CDH1* encodes E-Cadherin, a transmembrane glycoprotein situated at adherens junctions, whose physiological role is the establishment and maintenance of cell–cell adhesion in polarized epithelia. E-Cadherin is usually lost at an early stage of tumor development. It starts the process by inducing the expression of a mesenchymal phenotype and eventually leads to progression and dissemination [20,62,73]. The *HER2*/*ERBB2*/*NEU* gene, amplified in 15–35% of BC cancers [74,75], encodes a RTK that activates EMT [76] as well as the repair of radiation-induced DNA damage [77]. Another RTK, AXL, was reported to activate EMT and HR even in TNBC cell lines [78].

Mutations in the genes encoding the cytokine TGFß or downstream signaling components have been associated with dysfunction of the skeletal, muscular and/or cardiovascular systems as well as cancer predisposition. A polymorphism causing high circulating TGFß levels has been associated with invasive BC [79]. TGFß regulates epithelial cell growth during mammary gland morphogenesis in a hormone-dependent fashion. It plays dual roles as a tumor suppressor during BC initiation and a promoter of EMT through activation of the transcription factor ZEB1 during BC progression [34,64,80]. TGFß also plays dual roles in the DNA damage response. On the one hand, TGFß was reported to inhibit BRCA1-dependent DNA repair via SMAD3 [37] and diminish ATM expression via miR-181 [81]. On the other hand, it activates ATM signaling and DNA repair through reactive oxygen species (ROS) [35] and p53 [38], which prevents aneuploidies as well as gene amplifications [82].

Other genes, frequently mutated in hereditary and sporadic BC or showing altered expression, are *PTEN* and *BRCA1* [21,23,83,84]. The phosphatase PTEN primarily acts as a tumor suppressor via inhibition of the PI3K/AKT axis, thereby controlling expression of key EMT factors such as TWIST1 [83]. Though very much under debate [85,86], PTEN was also reported to act on chromatin in BC cells and to increase expression and recruitment of RAD51, thereby promoting HR and replication fork recovery [87,88]. All-in-all, a number of signaling pathways targeted by genetic changes in BC modulate both EMT and DNA damage responses.

#### 2.1.2. Crosstalk between Nuclear DNA Damage Response Components and EMT

The roles of BRCA1 in HR-mediated DNA repair have been attributed to coordinating CtIP-dependent end processing during the cell cycle [89] and stimulating RAD51-mediated homologous pairing [90]. During DNA replication, the BRCA1, BRCA2 and Fanconi anemia (FA) pathway components, such as FANCD2, protect regressed forks at DNA replication blocking lesions [13,14]. Concomitantly, BRCA1 antagonizes EMT directly and indirectly. Thus, BRCA1 was observed to exert an inhibitory effect on TWIST1 and FOXC1/2, another EMT-inducing transcription factor downstream of SNAIL and SLUG, by direct promoter binding [91]. The Livingston team made the exciting discovery that BRCA1 repairs crosslinks in complex with FANCD2, the SWI/SNF chromatin remodeling factor BRG1, the NOTCH target gene product HES1, and the differentiation maintenance factor NUMB. Failure causes DNA damage, which triggers the luminal to basal and mesenchymal transdifferentiation of mammary epithelial cells with a rise in EMT factors [10,36,47]. Curiously, such an aberrant differentiation could be recapitulated by Cisplatin treatment, i.e., chemical crosslink formation, but not Etoposide treatment, inducing primarily DSBs. Moreover, Chen et al. [92] noticed EMT in response to treatment with the anthracycline Doxorubicin, and Anandi et al. [93] in response to alkylation damage by *N*-methyl *N*-nitrosourea (MNU). Conversely, Yoshida et al. [94] found that the microtubule-modulatory drug Eribulin reverts EMT. In conclusion, persistent DNA replication stress could be a common denominator of EMT-induction, as the EMT-inducing chemical treatments do generate DNA replication blocking lesions, such as crosslinks [95,96,97]. Of note, BRCA1 defects cause accumulation of the same replication blocking lesions [18,47].

Further upstream, the adapter molecule RAP80 is involved in the recruitment of the BRCA1 repair complex to the damaged chromatin labeled by ATM- or ATR-mediated phosphorylation of histone H2AX [98]. There is evidence that RAP80 suppresses *ZEB1* expression, which may involve an unidentified miRNA [50]. A series of enlightening experiments strengthened by in silico analysis of human adenocarcinoma samples revealed that depletion of histone H2AX activates the EMT genes *SLUG* and *ZEB1* through accumulation of transcription activation marks H3K9ac and H3K27ac at their promoters. Re-expression of wild-type H2AX, but not a DNA repair-inactive phosphorylation site mutant, reverted the mesenchymal phenotype, coupling DNA repair with EMT suppression [99]. Thus, DNA repair components upstream of BRCA1 also exert an inhibitory effect on EMT transcription.

The tumor suppressor p53 plays a vital role in promoting error-free DNA repair and high-fidelity DNA replication [100,101,102,103,104]. Wild-type p53 induces the expression of miRNA200 (a-c) and miRNA34a, which suppress *ZEB1*, resulting in an inverse p53–ZEB1 expression pattern in human BC [55,105]. However, p53-induced miRNAs are subject to negative feedback regulation, because ZEB1 is able to bind to and repress the respective promoters [55,56,105]. Such a bimodal relationship has also been established between BRCA1 and EMT. Although BRCA1 inhibits EMT transcription factors TWIST1 and FOXC1/2 [91], SLUG and SNAIL were shown to suppress *BRCA1* expression by direct repression of the promoter and indirectly by recruitment of the chromatin-demethylase LSD1 [49]. Indeed, an inverse relationship between BRCA1 and the EMT marker Vimentin as well as cytoskeletal proteins typical of basal-like tumors, i.e., CK5/6, CK14, CK17, were observed in BC, and underscore the central role of BRCA1 in suppressing invasiveness and metastasis [91]. Altogether several DNA repair proteins antagonize EMT, but negative feedback loops towards BRCA1 and p53 serve as fine-tuning mechanisms to generate flexible switches to turn on EMT for migration and MET for metastatic growth [106]. These feedback loops may also explain why mRNAs encoding HR genes were found to be upregulated in brain metastases of BC patients, though mutations simultaneously rose in the same group of DNA repair genes, *BRCA1* in particular [74].

A different pattern is seen, when focusing on enzymes transducing DNA damage signals via post-translational modifications. Although the components of accurate DNA repair antagonize EMT, as outlined above, DNA damage response components activate EMT. First, the E3 ubiquitin ligase RNF8, and modifying histone H1 for recruitment of DNA repair proteins, also stabilizes SNAIL via the PI3K/AKT pathway and modifies TWIST1 for nuclear localization and transcriptional activity [107,108,109]. Second, PARP1, poly(ADP-ribosylating) a plethora of DNA repair and replication factors, stabilizes SNAIL via Integrin-linked-Kinase (ILK) and TGFβ pathway activation [61]. Third, PARP3 promotes TGFβ-induced EMT in BC after sensing ROS, proposedly as it establishes an appropriate chromatin configuration at responsive TGFβ genes [40]. Forth, ATR-CHK1 kinase signaling is activated by elevated NOTCH1 expression, which drives EMT and tumor progression. In this way BRCA1-deficient TNBC cells are protected from mitotic catastrophe by the restoration of S/G2 and G2/M cell cycle checkpoints [110]. Fifth, ATM phosphorylates and, thereby, stabilizes ZEB1 in response to DNA damage. ZEB1, in turn, deubiquitinates and stabilizes CHK1 via USP7; thereby, promoting HR [59]. Strengthening this observation, Prodhomme and colleagues observed a causal relationship between low ATM and low ZEB1 levels in TNBC cells [46]. Therefore, DNA damage response components involved in post-translational protein modifications exert a stimulatory effect on EMT.

Interestingly, Prodhomme and colleagues further noticed de-repression of the compensatory polymerase θ-dependent, mutagenic MMEJ backup pathway in basal-like, HR-deficient TNBC cells due to low ZEB1 expression [46]. Previous evidence for such a pathway shift was provided by separate analysis of DSB repair pathways in primary BC cells ex vivo [6]. This study revealed upregulation of MMEJ activity in cells displaying EMT and derived from BC patients with high-risk tumors. Along this line, recent data from CTCs of triple-negative MBC patients demonstrated a downregulation of 53BP1, which protects DNA ends from processing for error-prone DSB repair [111]. Manifestation of such mutagenic repair in TNBC was indeed demonstrated by comprehensive genomic analysis of BC subtypes engaging Next Generation Sequencing (NGS) [112]. A mutagenic escape mechanism may also exist in HER2+ BC brain metastases, which are devoid of pathogenic *BRCA1* mutations, but show a gene expression signature corresponding to BRCA1-deficient BC cells [113]. From this, we propose that mutagenic, compensatory DSB repair enables BC cells to cope with the damage that induces EMT [10,47].

All-in-all, a simple picture cannot be drawn of these complex relationships that are reshaped during the different stages of invasion, migration, and metastases. Yet, the high-fidelity DNA repair factors, such as BRCA1, seem to antagonize EMT, DNA damage sensors, and transducers like ATM, executing post-translational protein modifications that, instead, promote this process. One hypothesis that may reconcile these at first sight discrepant patterns relates back to the observations made by the Livingston team [10,47]. Accordingly, the damage itself, relayed by ubiquitination, phosphorylation, acetylation and poly(ADP-ribosylation) events and originating from DNA repair dysfunction, from ROS, accumulating due to enhanced mitochondrial oxidative phosphorylation (OXPHOS) [114] or from chemotherapeutic treatment, may represent a major driver of EMT. Upon the failure to remove this damage by high-fidelity DNA repair, reactivation of DNA damage responses and compensatory DNA repair may prevent toxic genome instability of invasive BC cells. We summarized the major pathways and interactions in Figure 1 and Table 1, accordingly.

### 2.2. Stemness and DNA Repair

Depending on the BC subtype a varying proportion of tumor cells display SC features, i.e., represent so-called cancer SCs or stem cell-like cells (CSCs). The CD44+/CD24^−/low^ marker set has been established best for identification of BCSCs [115]. This marker set differentially identifies percentages of ≤97% in basal-like BC as compared to only ≤17% in luminal BC, characterized by ER-positivity [116]. Basal-like BC, defined by a gene expression signature including strong expression of cytokeratins (CK) 5, 6 and 17, largely overlaps with TNBC, defined by the absence of ER, PR and of HER2 overexpression. Consequently, TNBC is as well enriched for tumor initiating cells, i.e., CSCs [19]. Given their unlimited capacity to self-renew, CSCs are drivers of tumor evolution and considered seeds of metastasis [11,116]. Even worse, DSB repair dysfunction, such as frequently observed in TNBC [21,23], can cause genomic instability in tumor initiating cells that transmit mutations to their progeny during tumor evolution.

CSCs were reported to display an increased DNA repair proficiency [117,118,119], which contributes to the genotoxic drug resistance of CSCs [11,116]. Recently, TNBC lines and tissues (Cancer Genome Atlas, TCGA, data base) were shown to activate the Frizzled 5 (FZD5)-Forkhead Box M1 (FOXM1) WNT signaling axis to transcriptionally upregulate the factors involved in stemness maintenance (e.g., ALDH1, CD133) and in DNA repair including BRCA1 [117]. A similar upregulation of DNA repair genes (e.g., *Ung, Chek1, Xrcc5, Brca1*) was observed in BCSCs from the mammary gland of *Trp53* knockout mice [120]. In conclusion, while TNBC is often characterized by DNA repair dysfunction of the BRCA1 pathway [21,23], a fraction of cells compensatorily upregulates DNA repair genes [117] and can even activate HR [121]. These cells, therefore, can develop chemoresistance on top of their metastatic potential. These results suggest strong and highly dynamic links between DNA repair activities and stemness features.

The BRCA1 protein influences stemness in multiple ways. First, it transcriptionally activates the NOTCH pathway, a key pathway leading to the differentiation of normal mammary cells [48]. Second, BRCA1 physically interacts with the SC factor c-MYC, to repress the transcriptional activity of this oncogene product [122], and third, binds the differentiation maintenance factor NUMB, to prevent DNA damage accumulation in differentiated cells [10]. The downregulation or inhibition of BRCA1 was demonstrated to enlarge the population of CSCs in TNBC [48,123]. A comparative inspection of breast tissue with and without *BRCA1* mutations revealed that it is necessary for the differentiation of ER-/ALDH1+ mammary SCs into ER+/ALDH1- luminal cells [124]. Therefore, BRCA1 is a negative regulator of stemness in BC cells.

There are other pathways that coordinate stemness and DNA repair. One critical pathway is the BMP/TGFß axis, which downregulates ATM, thereby, de-repressing stemness features in TNBC but not luminal cell lines [39]. In mammospheres from HER2+ expressing BC cell lines, ATM was reported to ensure the maintenance of CSCs by modulating the expression of autophagy, cell cycle and DNA repair genes [125]. These observations suggest a context-dependent influence of ATM on CSCs. The DNA repair factor PARP3 is highly expressed in basal-like BC, facilitates TGFβ-induced stemness and EMT and can shift DSB repair towards mutagenic non-homologous end joining (NHEJ) [40]. PI3K/AKT signaling enlarges the BCSC population through inhibition of the kinase GSK-3ß thereby stabilizing nuclear SNAIL [126,127]. BRCA1 is also controlled by phosphorylation through AKT1, excluding BRCA1 from the nucleus [128]. Of note, in BRCA1-deficient tumors, there is a constant activation of the PI3K/AKT axis [11,129].

Stemness features are regulated by several transcription factors, which are coupled with the EMT process. These factors are SNAIL, TWIST, SLUG and ZEB1/2, in particular. In agreement with the concept of fine-tuned mechanisms, they cooperate in a sophisticated manner. Although a high SLUG/SNAIL ratio facilitates stemness in basal epithelial cells, a shift towards a high SNAIL/SLUG ratio induces mesenchymal features and is involved in the generation of tumor-initiating cells [45,62,130]. Accordingly, SLUG promotes mammosphere formation, confers invasive and metastatic features and is primarily expressed in basal-like BC [62,131,132]. Although basal-like BC largely overlaps with TNBC, a SLUG-dependent shift to basal phenotypes has also been observed in non-TNBC, which correlated with treatment resistance [133]. Of note, TNBC can be subdivided into claudin-low BC enriched for mesenchymal and stemness markers CD44+/CD24- and basal-like TNBC characterized by stemness markers such as CD133 and MET [25]. The claudin-low subgroup of TNBC showed low chromosomal instability and low frequency of *TP53* mutations but varying degrees of claudin-lowness suggestive of a transient feature [134,135]. Basal-like TNBC includes *BRCA1* mutated tumors with high chromosomal instability and frequently mutated *TP53* [25]. Of note, BCSCs within the metastable EMT state can be subdivided into CD44+ mesenchymal BCSCs and ALDH1+ epithelial BCSCs, i.e., precursors of claudin-low and basal-like tumors [33]. The Wicha lab demonstrated that epithelial BCSCs perform enhanced OXPHOS and, therefore, are dependent on NRF2-mediated antioxidant defense programs. Quenching of ROS shifts these ALDH1+ epithelial BCSCs towards their CD44+ mesenchymal BCSC counterparts [136]. These examples illustrate the large toolbox endowing BCSCs with the genetic and phenotypic flexibility necessary for tumor evolution.

## 3. Dynamic Changes in the Regulatory Network of Stemness, EMT Features and DNA Damage Responses of CTCs

Circulating tumor cells have gained major interest as potential non-invasive diagnostic tools once their prognostic value for MBC patients was unequivocally demonstrated in large prospective studies [137,138]. Different from circulating tumor DNA, CTCs not only permit bulk genomic analysis of CTCs in the bloodstream, but also the identification of genetic changes in single CTCs and their potentially metastatic progeny [139,140]. Further, CTCs enable the detection of protein-based molecular markers in individual CTCs and an assessment of their functional status [115,139,141]. With the advent of synthetically lethal treatment strategies, phenotypic analysis of tumor cells has become an extremely promising approach to capture single and combined defects resulting from genetic or epigenetic mechanisms [142,143]. In recent years, major efforts were made to establish methods for the analysis of DNA repair functions in freshly isolated BC tissues [6,144] and CTCs from metastatic cancer patients [111,145,146,147,148,149]. Regarding the functional analysis of primary BC, so far, the most promising approach is immunofluorescence microscopy of RAD51 in S-phase nuclei of tumor slices ex vivo, which provides a biomarker for the response to PARP inhibitory drugs with superior predictive value as compared to a genomic signature correlating with HR-deficiency [150].

Regarding the functional analysis of CTCs, a pioneering study by the Trumpp team [151] demonstrated enrichment of EpCAM+ CTCs with positivity for the stemness markers CD44, CD47 and MET among metastasis initiating CTCs. Yet, the authors needed to xenotransplant at least 1000 CTCs from the luminal MBC patients into immunodeficient mice to detect metastasis, which unfortunately excludes such an in vivo method for biomarker development with blood samples from metastatic cancer patients with ≥1 CTC/7.5 mL in only 20–50% of the cases [137]. Efforts were made to overcome this limitation by (i) enrichment of CTCs from large blood volumes by apheresis [152,153]; (ii) ex vivo short-term culture of CTCs [141]; and (iii) establishment of permanent CTC lines as a model for the primary tumor [154,155].

For CTC enrichment and single cell isolation from blood samples, a large variety of methods were developed, namely based on the selection of CTCs via surface markers such as EpCAM (positive selection), via removal of leukocytes (negative selection), via immunocytochemical staining (flow cytometry, laser microdissection) and via filtration or microfluidic devices (size-based, deformability) (for overview see [115,156,157]). Most of todays’ knowledge of CTCs from MBC has been obtained from the molecular analysis of CTCs enriched after blood draws using fixatives [141]. Among these technologies analyzing fixed cells, the CellSearch System^®^ was approved by the FDA for diagnostic testing and, consequently, engaged in numerous prospective clinical trials in search for prognostic and predictive markers. The CellSearch System^®^ relies on immunomagnetic enrichment of EpCAM+ cells, immunostaining, and fluorescence microscopy to verify the epithelial cell origin (CK+, CD45-) and the assessment of additional markers of interest. However, a side-by-side analysis of EpCAM-dependent and -independent enrichment of CTCs indicated a significant downregulation of EpCAM in CTCs from triple-negative MBC patients [158]. Moreover, EpCAM-negative CTCs from MBC patients were found to be particularly invasive and competent in generating metastases in xenografted mice [159]. Results from other studies unveiled that more than 75% of CTCs from MBC patients co-express both epithelial (CK) and mesenchymal (Vimentin, N-Cadherin) markers, and half of them display SC markers CD44+CD24^−/low^ or ALDH1^high^CD24^−/low^ [115]. These and further data from ex vivo CTC cultures suggest a continuous transition between epithelial and mesenchymal features as well as changes in the degree of stemness [160].

### 3.1. Plasticity Causes Stress in CTCs

During their journey from the primary tumor to the distant metastatic site, CTCs undergo dramatic changes involving acquisition and a loss of stemness features, EMT and MET, detachment, migration and attachment, intravasation and extravasation, DNA damage responses to genotoxic stress, and genetic alterations conferring drug resistance [1,161,162,163]. Moreover, published evidence indicates that CTCs can induce systemic and localized inflammatory responses via functional neutrophil conversion, promoting metastatic seeding [164,165]. It was also reported that DNA damage in tumor cells and cell-free DNA released from dying tumor cells may induce inflammation; thus, amplifying the stress stimuli and metastatic potential [166]. All-in-all, even before cytostatic treatment, CTCs are exposed to mechanical stress transduced to the nucleus [63] as well as oxidative stress from metabolic reprogramming [167] and from immune cells producing a spectrum of ROS [166].

#### 3.1.1. Interplay between Stemness, EMT and DNA Damage Response Pathways in CTCs

To permit fast morphological and functional changes, CTCs adopt hybrid states with high plasticity between epithelial (e.g., EpCAM, E-Cadherin) and mesenchymal (e.g., SLUG, SNAIL) characteristics [62,156,168]. In this way, CTCs can cross from EMT to MET and vice versa creating a heterogeneous and evolving CTC population that associates with a metastasis initiating capacity. In fact, recent evidence showed that this intermediate phenotype with co-expression of both epithelial and mesenchymal markers is the one coupled with SC features and a prerequisite for tumorigenicity of BC cells [169,170]. Using a multiplex immunofluorescence assay, Savelieva et al. [171] analyzed CTCs from 38 patients with invasive BC demonstrating that all CTCs displaying the EMT marker N-Cadherin also presented stemness markers, yet of different subsets regarding CD44, ALDH1 and/or CD133 expression. Stemness (CD44+CD24-) was also detected in the absence of N-Cadherin and associated with lymph node metastasis. Very convincingly, Papadaki and colleagues [172] analyzed CTCs from 130 MBC patients for CSC (ALDH1) and EMT (nuclear TWIST1) markers by immunofluorescence microscopy. Their study provided evidence for an association between the CSC+/partial EMT status in 28% of the patients with lung metastases and reduced progression-free survival (PFS). Interestingly, the mean percentage of CTCs with this hybrid phenotype rose to 58% after taxane and/or anthracycline chemotherapy, but in non-responders only. From this and further work, it can be concluded that partial EMT in a subset of CTCs, with both epithelial and mesenchymal features, is connected with plasticity of stemness, formation of metastases after xenotransplantation in immunodeficient mice, and survival after chemotherapy [115].

Important insight into the rapid regulatory effects came from the analyses of CTC clusters with a 20–100 times greater metastatic potential than single CTCs [173]. Gkountela and colleagues [174] observed the hypomethylation of genes encoding stemness factors including OCT4, SOX2, NANOG and SIN3A upon the clustering of CTCs from BC patients, which was reverted upon the dissociation of the clusters into single CTCs. Cell–cell junctions, such as mediated by E-Cadherin, turned out to be required for the maintenance of this stemness phenotype, which was reminiscent of embryonic SCs. Moreover, enrichment of tumor-initiating BC cells by growth in mammospheres induced hypomethylation of several gene components of the JAK-STAT pathway, which was found to be constitutively active in putative CSCs (CD44+/CD24^low^). In support, hypomethylation of EMT and stemness genes, together with hypermethylation of epithelial marker genes, was as well detected in CTCs versus primary tumor cells from lung cancer patients [175].

BRCA1 transcriptionally represses basal-like cytokeratins, so that loss or inhibition of BRCA1 increases the subpopulation of BC cells with stemness markers like ALDH1 and improves spheroid growth [176]. These and further data [11] showed that BRCA1 controls multiple pathways to prevent the enrichment of SCs and the maintenance of stemness. Similarly, depletion of ATM or its substrate CHK2 were sufficient to induce sphere formation in BC cells [39]. On the other hand, DNA damage response and repair genes, including BRCA1, have been found to be upregulated in BCSCs of a mouse mammary gland tumor model [120] and in brain metastases of BC patients [74], suggesting that CTCs presenting SC features encounter conditions that require efficient removal of DNA lesions. In further support, Pieraccioli et al. [177] found induction of the DSB repair genes *XRCC2* and *XRCC4* by the EMT and stemness transcription factor ZNF281. Single CTC analysis by multiplex transcriptome profiling revealed subsets of CTCs within individual patients, suggesting a coupling between EMT and stemness features, and DNA repair proficiency such as via *RAD51* [178]. Along this line, Mani et al. [179] carved out an aberrant upregulation of GLI1 in BC from TCGA data, particularly in TNBC. The authors experimentally demonstrated, in TNBC lines, that GLI1, known to promote stemness and EMT phenotypes, upregulates FANCD2 transcriptionally; thereby, augmenting HR, replication fork protection, and PARP inhibitor resistance. Resistance to genotoxic treatment (Pirarubicin, Cyclophosphamide) was also true for the CTC-3 cell line, which correlated with high Vimentin, CD44 and low E-Cadherin expression [180]. Gong et al. [149] demonstrated that primary BC cells accumulate γH2AX-labeled DNA damage after detachment, but subsequent DNA repair was faster in suspension. CTCs, when attached, showed aggravated DNA damage accumulation suggesting augmented DNA repair in suspension. Engaging the MBC cell line MCF7 as a model, the authors further demonstrated an activation of DNA damage responses, namely elevated ATM, ATR, CHK1 and CHK2 kinase activities, upon detachment from the extracellular matrix and increased expression of ERCC1 and RAD51 in primary BC upon prolonged suspension culture. All-in-all it seems that the high degree of plasticity enabling CTCs to migrate entails genotoxic stress and, consequently, an adaptive DNA damage response.

#### 3.1.2. Sources of DNA Damage in CTCs

Where does this genotoxic stress stem from? One source could be mechanical stress, as the cytoskeleton undergoes dramatic changes during EMT. Evidence has accumulated showing that the intermediate filament Vimentin generates a network between the plasma and the nuclear membrane, which provides mechanical support, protecting the cells against nuclear rupture during migration [63,181,182]. It connects with the linker of the nucleoskeleton and cytoskeleton complex (LINC) and, therefore, with the nuclear lamina [182], whose integrity is essential to prevent nuclear blebbing, heterochromatin changes and DNA damage [183]. In this way, CTCs cope better with the mechanical challenges during intravasation at the primary BC site, circulation in the bloodstream and extravasation at the metastatic site [44,45]. Plasticity of the cell and the nuclear shape can be achieved by up- and downregulation of Vimentin limiting genome instability. Lorentzen and colleagues [162] made the fascinating observation that CTCs in patients form a cell pole composed of cytoskeletal proteins, i.e., ezrin and villin, F-actin, phosphor-myosin light chain and integrins, and this pole is required for attachment and metastatic seeding. Simultaneously, CTCs in circulation seem to have an increased capacity to repair DNA damage, including that caused by chemotherapeutic drugs. This shift seems to happen at the point of detachment from the stromal matrix [149]. These observations suggest that cytoskeletal proteins of CTCs are involved in triggering adaptive stress responses.

Another source of DNA damage in CTCs is oxidative stress. Zheng et al. [167] hypothesized that CTCs experience oxidative stress in the bloodstream when arriving from the hypoxic environment in the primary BC. Indeed, the authors demonstrate that intracellular ROS is elevated and counterbalanced by endogenous antioxidants in CTCs but not in primary BC, which prevents apoptosis in CTCs and permits metastasis. Schafer et al. [184] proposed that detachment of mammary epithelial cells from the extracellular matrix induces ROS due to reduced glucose uptake required for the antioxidant-generating pentose phosphate pathway. Glucose uptake could be restored by HER2 overexpression or via PI3K/Akt pathway activation, which can also be achieved by overexpressed MYC through the miR17-92 cluster [185]. Intriguingly, amplification of *HER2*, *MYC*, *CCND1* and *MDM2*, deletions of *PTEN*, low ER/PR expression and, more generally, an advanced stage of BC and poor prognosis have been associated with a high mitochondria content [186]. Suppression of ROS can also be mediated by the pluripotency factor *KLF4* transcriptionally upregulating ß-globin in CTCs [167]. Morel et al. [134] correlated ZEB1 expression in human mammary SCs with a protective antioxidant program driven by the methionine sulfoxide reductase MSRB3. Supporting data were obtained by molecular characterization of line CTC-MCC-41 from a colon cancer patient with metastasis initiating properties. The CTC-specific molecular signature indicated elevated mitochondrial energy production, fatty acid synthesis, proliferation, a stemness gene set, FA pathway components, and a concomitant rise in p53 signaling [187]. Given that p53 was reported to promote survival, metabolic reprogramming, and ROS clearance early after metabolic stress, this response may contribute to the quick adaptation to the new microenvironment of CTCs [188]. In summary, increased ROS result from the increased metabolic demand of the mitochondria in CTCs, displaying markers of CSCs [189,190], and are counterbalanced by various adaptive responses (see Section 3.2).

Another source of genotoxic stress in rapidly growing tumor cells, that is intertwined with oxidative stress, is oncogene-induced replication stress. Upregulation of MYC, the prime example of an oncogene product, via WNT/ß-catenin signaling has been observed in response to an increase in ROS at the invasive front of BC enriched in BCSCs [185]. Of interest, *MYC* alterations were described in 62% of CTC+ patients [191]. CTC-specific *MYC* amplifications promote stemness and create a selection bias for metastasis; they were detected in seven out of nine BC cases by Gao and colleagues [2]. Physiological levels of MYC control the activity of G1 cyclin-dependent kinases [192] and coordinate transcription with DNA replication and cell cycle progression, limiting transcription-replication conflicts [193,194]. Excess MYC drives the cell into rapid cell cycle divisions, exacerbating multiple sources of endogenous replication stress [189].

Mechanistically speaking, all mentioned types of challenges, i.e., mechanical, oxidative, oncogene- and chemotherapy-induced stress merge at the replication fork, i.e., cause replication stress. Moreover, rapidly dividing tumor cells are devoid of proper checkpoint control, frequently show dysregulated origin firing, replication-transcription conflicts, and exhaustion of the deoxynucleotide (dNTP) pool [189]. Therefore, BC cells have developed mechanisms protecting against replicative stress. One such mechanism is driven by SLUG, which is important for EMT, SC biology and the metastasis of BC cells [62]. Following a replication block it activates ATR-CHK1 DNA damage response signaling via the single-stranded binding protein RPA32 covering the resected DNA strands [62,195]. In recent years, translesion DNA synthesis has gained interest as an important mechanism alleviating replication stress and creating resistance to PARP inhibitors and Cisplatin in BC cells [189]. Feng and colleagues [196] found that knockdown of the translesion synthesis polymerase ζ subunit REV7 inhibits BC cell migration and invasion. As translesion synthesis bypasses DNA damage, but itself is mutagenic [197], translesion synthesis could be a mechanism for a CSC to balance survival and mutagenesis under genotoxic stress [198]. In this context, it is of interest that recent work from our lab discovered a novel DNA damage tolerance mechanism that is mediated by p53 in complex with the translesion synthesis polymerase ɩ [100]. Of interest when studying CTCs that are considered tumor-initiating or CSC-like [190], we found a critical biological impact of this novel DNA damage tolerance pathway in tumor-initiating cells from ovarian cancer xenografts. More specifically, we observed dual roles of polymerase ɩ and p53 in decelerating DNA replication by ZRANB3-mediated fork reversal for bypassing replication barriers in CSCs, but fast and mutagenic translesion synthesis in more differentiated cancer cells [100]. Underscoring the impact of this finding for CTCs from BC patients, elevated expression levels of polymerase ɩ were reported for BC tissues, associated with lymph node metastasis and to promote migration and invasiveness of BC cells [199]. Consistent with this model, MBC cell lines and CTCs survived better when *TP53* was the wild-type after PARP inhibitor treatment, while DSB-inducing treatments caused cell killing [154,200]. Altogether, CTCs encounter a large spectrum of genotoxic challenges both caused by and mastered through their highly plastic states.

### 3.2. Evidence for CTC-Specific DNA Damage Responses and Their Manifestation at the Genomic Level in Breast Cancer Patients

#### 3.2.1. Accumulation of Genomic Instabilities in CTCs from Breast Cancer Patients

A comparison of multiple tumor types showed that BC and ovarian cancer are the tumors driven by CNAs rather than point mutations found in colorectal carcinomas [201]. Moreover, CTCs from BC patients also harbor CNAs (Table 2). This genetic make-up can be explained by the inactivation of genes such as *BRCA1*, *BRCA2* and *TP53* [201], and the resulting HR dysfunction with a rise of aberrant pathways [6,7]. Chromosomal structural changes such as gene amplifications are triggered by DNA replication problems and/or genomic DSBs [202]. The molecular analyses of copy number changes and their borders led to different models that can explain their genesis. According to the first one, they can start from genomic DSBs, which are repaired by error-prone HR between different alleles (non-allelic HR) or by single-strand annealing. More recent models rely on replicative mechanisms that are initiated at stalled or collapsed, i.e., broken replication forks. These mechanisms involve the replication of non-contiguous DNA segments either by template switching or microhomology-mediated break-induced replication. Both models underscore the impact of the functionality of DSB repair and DNA replication machineries in preventing CNAs and, therefore, BC.

Of note, Nadal and colleagues [191] observed CNAs in primary BC specimens, i.e., at early stages of MBC, correlating with CTC release in 14 cases (Table 2). Engaging EpCAM-independent enrichment protocols, Riebensahm and colleagues [158] demonstrated that CTCs were detectable in 70.0% of the subgroup of TNBC patients as compared with 32.6% of all investigated patients with divergent BC subtypes (*n* = 46). Intriguingly, TNBC frequently features BRCA1 pathway dysfunction [21,23]. Inspired by these observations, we propose that BC with compromised replication fork protection and/or deregulated DSB repair will accumulate CNAs, which will accelerate CTC release. *MYC* amplification, among other CNAs, is one reasonable candidate to empower CTCs to cope with the challenges during their journey from the primary tumor to the distant metastatic site (see Section 3.1). Thus, MYC expressing cells can acquire CSC features and induce EMT in breast epithelial cells [206]. Moreover, overexpression of MYC causes DNA damage during S-phase [193], spurring further chromosome aberrations during the evolution of CTCs.

Gao and colleagues [2] identified CNAs by whole-genome amplification (WGA) and NGS in 23 patients. Their data indicate gradual accumulation of CNAs from single primary BC cells to CTCs. A detailed analysis of the breakpoints revealed complex rearrangements combined with gene amplifications as the underlying mechanism. The authors concluded that microevolution of genomic rearrangements gives rise to driver mutation-specific CNAs such as MYC amplification. Driver mutations will enable the transformations necessary to challenge genomic stability, mobilize tumor cells and cross the blood–brain barrier in BC patients with brain metastases. Therefore, CTCs are subject to selection pressures that can explain the high clonality of CTCs as was observed by Riebensahm et al. [158] in MBC patients. Altogether, these data strengthen the concept of mutational events driving BC development starting from the earliest stages followed by the selection and outgrowth of genetically altered cells during the later stages of distant progression [202].

The team around Christoph A. Klein pioneered the analysis of chromosomal imbalances in single tumor cells during BC progression using comparative genomic hybridization (CGH) as well as PCR-based analysis of loss-of-heterozygosity (LOH) and of *HER2* amplification [207]. The authors observed that a subset of their BC patients (*n* = 47) featured early chromosomal changes, which was detectable in at least a fraction of the primary tumor cells as well as in disseminated tumor cells in the bone marrow. Yet, the majority of *HER2* amplifications emerged late during tumor cell evolution. In agreement, Meng and colleagues [203] used fluorescence in situ hybridization (FISH) to monitor *HER2* amplification in the primary tumor and in CTCs. Their data indicated acquisition of this genetic change in 37.5% (9/24) of *HER2* amplification-negative BC patients during advanced stages and intensive treatment with radio- and/or chemotherapy. Monitoring HER2 immunocytochemically using the CellSearch System^®^ revealed CTC-specific acquisition of HER2 overexpression in 18% of patients (8/45) with advanced BC undergoing anti-HER2 trastuzumab treatment [208]. The genomic disparity between primary BC cells and CTCs was confirmed by molecular characterization of CTCs from 66 patients after the WGA of individually isolated cells [139]. Whole-genome analyses of circulating tumor microemboli, i.e., clusters of CTCs shed from early-stage BC (*n* = 6), identified 30–63% of private alterations in CTCs already at that stage [204]. Following a similar protocol for the NGS-analysis of CTCs, Paoletti and colleagues [161] investigated resistance mechanisms to endocrine therapy that are known to arise in nearly all ER/PR+ MBC patients. An analysis of mutations and CNAs in 130 genes in single and pooled CTCs from 11 patients with ER/PR+ MBC revealed various mechanisms of resistance. Most prominently, acquisition of activating *ESR1* mutations or CNAs were found in four patients. At the same time, the authors observed a concordance of 85% in at least one of the key somatic mutations and CNAs between the paired CTCs and metastatic tissue. Other key genes showing such concordance in more than one patient were *TP53* (6/11), *PIK3CA* (6/11), *MYC* (3/11), *CDH1* (2/11) and *CCND1* (2/11). Altogether, these findings supported the concept that an accumulation of mutations continues during tumor progression and treatment [209], giving rise to CTCs and, ultimately, metastases with new targets for treatment.

#### 3.2.2. CTC-Specific DNA Damage Responses of Breast Cancer Patients

Each human cell is subject to ~70,000 DNA lesions per day, whereby the majority of the lesions (70–80%) are single-stranded DNA breaks, which can arise from oxidative damage and are converted to DSBs when encountered by the replication fork [210,211]. Another DNA lesion generated upon exposure to oxidative stress is the base modification 7,8-dihydro-8-oxo-guanine (8-oxo-G), which is found at levels of 10^3^ lesions per cell/per day in normal human tissues, rising to 10^5^ lesions per cell/per day in cancer tissues [212]. This DNA lesion is highly mutagenic in several types of tumors including BC and ovarian cancer. It results in C:G to A:T transversion mutations, possibly via error-prone bypass engaging translesion synthesis polymerase ɩ [213]. The increase in intracellular ROS can be explained by the activation of oncogenes or loss of tumor suppressor genes entailing high metabolic activity and mitochondrial dysfunction (see Section 3.1).

Gong et al. [149] detected intracellular ROS and 8-oxo-G in BC cells using fluorescent dyes and immunofluorescence microscopy, respectively. Their study led to the interesting observation that ROS signals are significantly higher in CTCs than in primary BC cells from the same MBC patients. Consistently, basal DNA damage was elevated in CTCs as was determined by nuclear signals of γH2AX. DNA repair was much faster in CTCs than in primary BCs in ex vivo culture, as judged from the removal of chemotherapeutic treatment-induced DNA damage labeled by γH2AX or DSBs indicated by neutral comet assay. This DNA repair in CTCs was sensitive to inhibition of the DNA damage response kinases CHK1 or CHK2. When primary BC cells were raised in suspension rather than adherent culture, DNA damage removal was also significantly enhanced. Vice versa, basal DNA damage in CTCs was aggravated by adherent culture on an extracellular matrix. Moreover, ROS damage in CTCs was reported to be counterbalanced by elevated expression of antioxidant factors such as thioredoxin or ß-globin [149,167]. As would be expected from the accelerated repair and antioxidant defense in CTCs, freshly isolated primary BC cells from 55 MBC patients before and after four cycles of chemotherapy showed a 3.5-fold increase in apoptosis but only a 1.6-fold increase in matching CTCs, whereby the increase in primary BC cells was limited to the ones from chemotherapy responders with partial remission or stable disease [149]. An increased resistance of CTCs was not related to an increased expression of the ABC reporter responsible for drug efflux. Rather, DNA damage responses and the removal of chemotherapy-induced DNA damage seemed to be pre-activated by basal oxidative stress, as concluded by the authors from their ex vivo DNA repair analyses of CTCs in the presence or absence of ROS quenchers. In conclusion, increased endogenous ROS in CTCs leads to adaptive changes, namely antioxidant protein expression and accelerated DNA repair, that could play key roles in metastasis and resistance to radiation and chemotherapy.

Koch et al. [155] succeeded in the establishment of an ER+ and PR+ CTC line from a MBC patient with a wide spectrum of CNAs, carrying a pathogenic *TP53* mutation and a deleterious change in *ATM*, indicating severe DNA damage response defects in these CTCs. However, reflecting the dynamic changes of CTCs after release from the primary BC, Paoletti et al. [161] found CNAs in the DSB repair genes *ATM*, *BRCA1-Associated Protein 1* (*BAP1*) as well as somatic mutations in *BRCA2* in CTCs, but no longer in metastases of the same patients. This observation matches the observed rise of various DNA repair factors in brain metastases as compared to primary BC [74,214,215,216] and underscores the need to monitor the DNA damage response status in real-time, i.e., phenotypically rather than genetically. It is tempting to speculate that the proteins XRCC4 and ERCC1, upregulated in MBC with key roles in the error prone DSB repair pathways NHEJ and SSA, compensate for the general BRCA1 pathway dysfunction in MBC [113,214,215]. Kasimir-Bauer et al. [205] did not detect significant changes in *ERCC1* mRNA expression analyzed by multiplex RT-PCR of EpCAM-enriched CTCs in patients before and after neoadjuvant therapy. Yet, these data are reminiscent of previous inconclusive results on ERCC1 expression in lung cancer, explainable by the fact that only one of the four *ERCC1* isoforms functions in the repair of platinum drug-induced interstrand crosslinks [217,218].

Aiming at the development of pharmacodynamic markers based on DNA damage responses, the team around Robert Kinders and James Doroshow [145,147] successfully established a semiquantitative assessment of γH2AX signals in CTCs from advanced cancer patients using the CellSearch System^®^. Among the patients enrolled in phase I clinical trials of investigational agents, three BC patients, including a *BRCA2*-mutated TNBC patient, were treated with Cyclophosphamide and PARP inhibitors (e.g., Veliparib). Regardless of the relative CTC numbers, all three BC patients showed a rise in the percentage of γH2AX+ CTCs on day 2 post-treatment, from as low as 0% to up to 64%, and in two out of the three cases, a subsequent drop down to baseline on day 5 post-treatment. Recently, our team provided evidence that CTCs display dynamic and treatment-inducible DNA damage responses during chemotherapy of MBC patients with Eribulin [111]. In this study, we included 67 MBC patients with HER2-negative CTCs in the DETECT trial program before, during and after chemotherapy. Engaging the CellSearch System^®^ we monitored nuclear signals of the DNA damage response protein 53BP1 in CTCs. We focused on 53BP1, as the DNA end-binding protein 53BP1 is a well-known antagonist of DNA end resection and, thereby, of compensatory, error-prone DSB repair pathways in HR-defective tumor cells such as *BRCA1*-mutated TNBC [219,220]. This explains why reduced 53BP1 expression is a mechanism of resistance to platinum-based compounds and PARP inhibitors. Moreover, 53BP1 requires intact links between cytoplasmic microtubules and the nuclear envelope via the LINC complex to promote roaming of DNA ends for repair, which made it a good candidate to monitor DNA damage by the microtubule inhibitor Eribulin [221]. Indeed, comparison of 53BP1 from CellSearch System^®^-based immunocytochemistry and genomic integrity scores from single cell WGA and PCR [139] showed that 53BP1+ CTCs are characterized by low genomic integrity. A longitudinal analyses showed that CTCs from triple-negative MBC patients displayed hardly any 53BP1 signals, whereas CTCs from patients with ER/PR + metastases showed an increase in nuclear 53BP1 signals with treatment. Kaplan–Meier curves revealed an increase in PFS with 53BP1-positivity after treatment, suggesting that the 53BP1 labelling of CTCs might serve as a marker of chemotherapeutic responsiveness of MBC patients.

## 4. Conclusions and Future Perspectives

Dynamic changes during tumor evolution enable MBC cells to escape from being killed by chemotherapeutics. First, DNA repair defects generate genetically unstable primary tumor cells that can give rise to resistant MBC through the adaptive selection of pre-existing genomic aberrations [222]. Second, altered DNA damage responses are not limited to the primary tumor, i.e., can continue to mutate the genome of CTCs. Third, differentiation and morphology changes, which are coupled with the journey to distant sites, modulate the DNA damage responses of CTCs in a highly dynamic fashion (Figure 1, Table 1). Of note, chemotherapeutic treatment itself can alter EMT and stemness, thereby increasing the spectrum of escape mechanisms [223]. Finally, CTCs can induce systemic and localized inflammatory responses via functional neutrophil conversion, promoting metastatic seeding [164,165]. DNA damage in CTCs and cell-free DNA released from dying CTCs can induce inflammation; thus, amplifying the metastatic potential [166]. For these reasons, Meng and colleagues [203] questioned approaches that engage the primary tumor when making treatment decisions during metastatic progression. So far, CNAs and HR signatures obtained from the genome of primary BC cells have been explored to predict PARP inhibitor responses [8,224]. Although they provide a snapshot from the past, molecular and functional analyses of CTCs from MBC patients capture the disease status in real-time.

To overcome treatment resistance, combination therapies were designed according to the principle of synthetic lethality. In particular, inhibitors of PARP1 together with the DNA damage response kinases ATR, CHK1 or WEE1 are being investigated in clinical trials [225]. Dual inhibitory molecules, co-targeting PARP1 and Bromodomain 4 (BRD4), which executes key functions in multiple processes including DNA damage responses, or PARP1 and RAD51, the key recombinase in HR, have been found to sensitize MBC cell lines regardless of hormone receptor status, BRCAness, or acquired resistance [226,227]. Suggesting the validity of these concepts for the eradication of CTCs, Gong and colleagues [149] demonstrated that chemoresistance due to the enhanced DNA repair in CTCs versus primary BC can be broken by adding inhibitors of the DNA damage response kinases CHK1 or CHK2. Given that EMT and stemness marker expression in CTCs was found in a much lower percentage of responders than non-responders of MBC patients [228], it is tempting to speculate that the combined or dual inhibition of EMT and stemness, and DNA repair components, will show synergistic effects on CTCs. Indeed, in TNBC cells, MYC blockade shows synthetic lethality with PARP inhibition [229]. Moreover, DSBs, arising in cancer cells during replication stress and/or in response to radio- or chemotherapy, were found to trigger STAT-IRF1 signaling; thereby, upregulating PD-L1 [230]. This pathway requires the exonuclease EXO1-dependent DNA end resection and is enhanced in HR-deficient cells. Expression of PD-L1 is detectable on CTCs from different cancer patients [231], so that immune checkpoint therapies provide another option for treatment of genetically unstable and aggressive tumors, such as TNBC.

Accumulating evidence showed that key HR proteins, like BRCA1, BRCA2 and other components of the FA pathway, exert replication fork stabilizing functions that can be separated from their canonical functions in HR [232]. DNA replication has traditionally been a target of anticancer therapeutics, directly in the case of antimetabolites and indirectly through lesions blocking DNA replication fork progression [225]. Though lesion bypass mechanisms involving replication fork reversal or translesion synthesis confer resistance to chemotherapeutics, the power of direct interference with these so-called DNA damage tolerance mechanisms has been recognized just recently [233]. Interestingly, a genome-wide CRISPR screen discovered synthetic lethal interactions between FA gene defects and depletion of the translesion synthesis polymerase ɩ or of the kinase CDK4 [214]. Polymerase ɩ shows elevated expression in BC, leading to a reduction in DNA replication fidelity [234]. Polymerase ɩ was identified as the key molecule for a DNA damage tolerance pathway choice in embryonic SCs, hematopoietic SCs, and ovarian CSCs [100]. CDK4, target of inhibitory drugs such as Ribociclib for ER/PR+ MBC patients, drives G1/S cell cycle transition and S-phase progression and seems to be required for translesion synthesis regulation. Translesion synthesis itself mediates resistance to platinum and PARP inhibitory drugs [235]. More specifically, depletion of the translesion synthesis polymerase ζ subunit REV7, linked with invasiveness, enhances sensitivity of BC cells to PARP inhibition [196,236], and expression of translesion synthesis polymerase η in BC protects against inter-strand crosslinking agents [237] and contributes to Cisplatin resistance of ovarian CSCs [238]. Altogether, DNA damage tolerance mechanisms are at the core of chemoresistance mechanisms in MBC and have been recognized as a potential ‘Achilles heel’ of CSCs. In conclusion, we propose that both altered DNA damage response and DNA damage tolerance mechanisms in CTCs from MBC patients convey therapy resistance and, therefore, are promising novel targets to be exploited in the future.

## Figures and Tables

**Table 1:** Effectors and targets of the signaling network connecting DNA damage responses and epithelial-mesenchymal transition (EMT) in breast cancer (BC).

Effector/Effector Group	Target/Group of Targets	References
**RTK**	SLUG/SNAIL/TWIST1/ZEB1	[34,35]
**NOTCH**	SLUG/SNAIL/TWIST1/ZEB1	[36]
**TGFß**	BRCA1/FANCD2	[37]
**TGFß**	p53	[38]
**TGFß**	ATM/ATR	[35,39]
**TGFß**	PARP3	[40]
**TGFß**	SLUG/SNAIL/TWIST1/ZEB1	[41]
**SLUG/SNAIL/TWIST1/ZEB1**	TGFß	[34]
**E-Cadherin**	TGFß	[42]
**SLUG/SNAIL/TWIST1/ZEB1**	E-Cadherin	[34]
**Vimentin**	TGFß	[42]
**SLUG/SNAIL/TWIST1/ZEB1**	Vimentin	[43,44,45]
**SLUG/SNAIL/TWIST1/ZEB1**	polymerase θ	[46]
**BRCA1, FANCD2**	SLUG, SNAIL, TWIST1, ZEB1	[47]
**BRCA1, FANCD2**	NOTCH	[48]
**SLUG/SNAIL/TWIST1/ZEB1**	BRCA1/FANCD2	[49]
**RAP80**	SLUG/SNAIL/TWIST1/ZEB1	[50,51]
**p53**	SLUG/SNAIL/TWIST1/ZEB1	[52,53,54]
**SLUG, SNAIL, TWIST1, ZEB1**	p53	[34,55,56]
**H2A.X**	SLUG/SNAIL/TWIST1/ZEB1	[57]
**RNF8**	SLUG/SNAIL/TWIST1/ZEB1	[58]
**ATM/ATR**	SLUG/SNAIL/TWIST1/ZEB1	[34,41,59,60]
**SLUG/SNAIL/TWIST1/ZEB1**	ATM/ATR	[45,59,60]
**SLUG/SNAIL/TWIST1/ZEB1**	CHK1	[34,59]
**PARP1**	SLUG/SNAIL/TWIST1/ZEB1	[61]
**PARP3**	SLUG/SNAIL/TWIST1/ZEB1	[40]

**Abbreviations**: ataxia telangiectasia mutated, ATM; ataxia telangiectasia and Rad3-related protein, ATR; breast cancer associated 1, BRCA1; checkpoint kinase 1, CHK1; epithelial-mesenchymal transition, EMT; Fanconi anemia complementation group 2, FANCD2; H2A histone family member X, H2A.X; microhomology-mediated end joining, MMEJ; poly(ADP-ribose)polymerase, PARP; receptor associated protein 80, RAP80; RING finger protein 8, RNF8; tyrosine kinase receptor, RTK; transforming growth factor ß, TGFß; zinc-finger transcription factor 1, ZEB1.

**Table 2:** Genomic instability and DNA damage responses in CTCs from BC patients.

Effect in CTCs	Observations	References
**CNAs in primary BC correlate with CTC numbers**	Copy number alterations (CNAs) in BC specimen of CTC-positive cases.	[191]
**CNAs rise with invasiveness**	CNAs differ between CTCs from individual patients but not between CTCs from same patient. CNA numbers increase from patients with ductal carcinoma in situ (DCIS) to patients with invasive ductal carcinoma.	[2]
**Clonality of CTCs in MBC patients**	NGS reveals high genomic clonality in CTCs from BC patients with brain metastases.	[158]
***HER2*** **amplification acquired**	Fluorescence in situ hybridization (FISH)-based detection of *HER2* amplification provides evidence for acquisition in 37.5% of BC patients during progression and/or treatment.	[203]
**Microevolution of genomic rearrangements**	Genomic disparity between primary BC and single CTCs detected by NGS; driver mutation-specific rise of CNAs.	[139,204]
***ESR1*** **mutations acquired**	Activating *ESR1* mutations in CTCs from MBC patients after endocrine therapy; 85% concordance between key mutations and CNAs in CTCs and metastases.	[161]
**Increased oxidative stress**	Intracellular ROS is elevated and counterbalanced by endogenous antioxidants in CTCs but not primary BC or MBC, which prevents apoptosis and permits metastasis.	[149,167]
**Potentiated DNA repair confers chemoresistance**	γH2AX-marked basal DNA damage is elevated in CTCs versus attached BC cells and partially activates DNA damage responses. Comet assay- and γH2AX-marked DNA damage induced by cytostatics (Epirubicin, Cisplatin) is repaired faster in CTCs vs. attached BC cells irrespective of BC stemness.	[149]
**γH2AX monitors response to DNA damaging drugs**	γH2AX signals accumulate in CTCs from BC and other patients after combined cyclophosphamide and PARP inhibitor treatment (Phase I).	[145,147]
***ERCC1*** **expression before and after chemotherapy**	*ERCC1* mRNA expression analyzed by multiplex RT-PCR of separated CTCs shows expression in 60–70% of patients before and after neoadjuvant therapy.	[205]
**CNAs coupled with DNA repair gene alterations in CTC line**	ER+ CTC line from MBC patient with wide spectrum of CNAs carries pathogenic *TP53* mutation and predicted deleterious change in *ATM.*	[155]
**53BP1 associates with chemotherapy response**	53BP1 accumulates in CTCs from MBC patients with hormone receptor-positive metastases and in Eribulin-responsive patients.	[111]
